# A Review of the Efficiency of White Light (or Other) Emissions in Singly and Co-Doped Dy^3+^ Ions in Different Host (Phosphate, Silicate, Aluminate) Materials

**DOI:** 10.1007/s10895-023-03250-y

**Published:** 2023-04-27

**Authors:** Leelakrishna Reddy

**Affiliations:** Department of Physics, University of Johannesburg, Johannesburg, South Africa

**Keywords:** Luminescence, Phosphor, Dy^3+^ ion, Transitions, Doped materials

## Abstract

In this review we will present several research papers pertaining to white colour (or other) emission from Dy^3+^ doped and undoped phosphor materials. The search for a single component phosphor material that could deliver high quality white light under UV or near UV excitation is an area of active research for commercial purposes. Amongst all rare earth elements Dy^3+^ is the only ion that could deliver simultaneously blue and yellow light under UV excitation. In optimizing the Yellow/Blue emission intensity ratios, white light emission can be realized. Dy^3+^ (4f^9^) displays approximately 4 emission peaks at around 480 nm, 575 nm, 670 and 758 nm corresponding to transitions from the metastable ^4^F_9/2_ state to various lower states, such as ^6^H_15/2_ (blue), ^6^H_13/2_ (yellow), ^6^H_11/2_ (red) and ^6^H_9/2_ (brownish red), respectively. In general, the hypersensitive transition at ^6^H_13/2_ (yellow) is electric dipole in nature and becomes prominent only when Dy^3+^ ions are positioned at low symmetric sites with no inversion symmetry in the host matrix. On the other hand, the blue magnetic dipole transition at ^6^H_15/2_ becomes prominent only when Dy^3+^ ions are positioned at highly symmetric sites in the host material with inversion symmetry. Despite the white colour emission from the Dy^3+^ ions, these transitions are mainly associated with parity forbidden 4f -4f transitions, the white light produced maybe diminished at times, hence the need to include a sensitizer to bolster the forbidden transitions experienced by Dy^3+^ ions. In this review we will focus on the variability of the Yellow/Blue emission intensities in different host materials (phosphates, silicates, and aluminates) from Dy^3+^ ions (doped or undoped) by studying their photoluminescent properties (PL), their CIE chromaticity coordinates and correlated colour temperature (CCT) values for white colour emissions that is adaptable to different environmental conditions.

## Introduction

White light-emitting diodes (WLED) is the next generation of solid-state lighting (present day use) that has replaced the traditional incandescent and fluorescent lamps of bygone days [1, 2]. The reason for its use is because of its superior brightness, long lifetimes, absence of toxic mercury and cost saving energy properties [1, 2]. There are essentially three ways to fabricate WLEDs: (i) a combination of a blend of red, blue, and green phosphor materials, (ii) a dual combination of a blue emitting light diode with a yellow emitting phosphor material, where the most famous Ce^3+^ doped yttrium aluminum garnet (YAG: Ce^3+^) commercial phosphor material comes to mind, and finally (iii) fabrication of single phased all inclusive phosphor material doped with specific rare-earth ions for colour tuning [1, 3]. From case (ii) above, a similar combination of colours to obtain white light can be achieved by doping Dy^3+^ ion in a single phased host material, because the characteristic emission colour for this Dy^3+^ ion lies in the blue and yellow colour regions of the electromagnetic spectrum.

Trivalent dysprosium Dy^3+^ (4f^9^) generally possesses 2 intense characteristic emission peaks at 485 nm, which corresponds to a blue magnetic dipole ^4^F_9/2_ → ^6^H_13/2_ transition and usually, a hypersensitive yellow electric dipole ^4^F_9/2_ → ^6^H_15/2_ transition at 575 nm, respectively. The optimal yellow to blue (Y/B) intensity peak emission ratios yields white light. The position of the Dy^3+^ ions in the host environment is of crucial importance for efficient white light emissions. When Dy^3+^ ions are positioned at low symmetric sites in the environment of the host material with no inversion center, yellow emissions at 485 nm are of relatively higher intensity compared to blue light emissions at 575 nm wavelength are observed. On the other hand, when Dy^3+^ ions are positioned at highly symmetric sites in the environment of the host without inversion symmetry, then blue light colour emissions are relatively higher than the yellow light colour emissions.

A host on its own by its very nature is unable to emit white light, thus the necessity for the embedding of dopant ions, which in this case is Dy^3+^ ions [1]. The search for a single phased phosphor material for white light emission for LED application when doped with Dy^3+^ ions or co-doped with other rare-earth ions (other colours such as blue and green) for persistent luminescence under UV excitation is an area of active research. There is potential need to fabricate materials with unconventional emission properties, where previously the existence of defects was found to suppress the photoluminescence properties of materials, but now defects are found to be a storage or a trapping site for charges in the generation of persistence luminescence that was observed with specific host materials [3]. Potential host materials that could fit this criterion could be phosphates, silicates, aluminates, borates, etc. However, white light emissions from Dy^3+^ doped host materials suffer some drawbacks due its parity forbidden nature of its 4f – 4f transitions of the Dy^3+^ ions.

In the later part of this review, an attempt is made to ameliorate this situation by co-doping it with other rare –earth ion to bolster the parity forbidden nature of the Dy^3+^ ions. However, this process may lead to different colour emissions (besides white colour light), depending on whether the dopant (Dy^3+)^ is a sensitizer or an activator, but the result is for prolonged luminescence emissions long after the UV excitation source is removed.

In this review, we will focus on research papers, firstly for enhancing white light emissions through Dy^3+^ doping, and in the second part, a look at persistent luminescence when Dy^3+^ is co-doped with other rare-earth ions in different host materials, as well as the mechanism for persistence luminescence will be discussed.

## Factors for Consideration for White Light Efficiency and Persistent Luminescence in Singly doped Dy^**3+**^ and Co-Doped Host Materials

Researchers in this review have published many papers using Dy^3+^ ions for white light emissions or co-doped it for persistent luminescence, but have not reached the desired standards for commercialization, due to degradation of the light or lack of consistency for prolonged luminescence (or the inefficiency of the mechanism).

Some, if not all these factors need to be factored in producing optimal emission characteristics (white light efficiency in the first instance and consistency of its illumination in the second instance) [4, 5]:

- Low preparation costs.
- High CRI and low CCT values.
- High chemical and thermal stabilities.
- Appropriate choice of host compounds and dopants.
- Nature of Y/B intensity ratios in producing electric dipole or magnetic dipole transitions.
- Emission lifetimes: fluorescence or phosphorescence emissions.
- Influence of concentration on the Y/B ratio in Dy^3+^ doped materials.
- Mechanism of white light colour emission from Dy^3+^ doped host materials.

### Low Preparation Costs

In advancing the use white LEDs or materials for prolonged luminescence, facile methods of preparation at low cost are necessary. Typical methods of preparation at a relatively low cost used by many researchers are the solid-state reaction and the combustion methods of synthesis. Single phased phosphor materials as opposed to the use of tricolor phosphor materials is a cost saving procedure in producing desired phosphor materials. In the case of aluminates and silicates, synthesis method play an important role in persistent luminescence, therefore a variety of synthesis methods were employed.

### High CRI and Low CCT Values

According to Wikipedia [November 2022], the colour rendering index (CRI) is “a quantitative measure of the ability of a light source to reveal the colours of various objects faithfully in comparison with a natural or standard light source”. This means that a light source must depict the natural colour of an object [4]. CRI values range from 0 to 100, thus a value of 100 implies that a light source is identical to the spectrum of daylight. In general, light sources should have CRI values greater than 80 for it to be commercially acceptable. The other factor for consideration is the correlated colour temperature (CCT) appearance. This is defined as the temperature appearance of a white LED light source (lamp). It is measured in kelvins and is determined using McCamy’s empirical formula: CCT = -449n^3^ + 3525n^2^ -6823n + 5520.33, where n represents the inverse slope line and is given by n = (x – 0.332)/ (y – 0.186). In general, CCT values fall in 4 categories: (2700 – 3000 K) is regarded as warm white light, (3000 – 4500 K) is regarded as bright white light and (4500 – 6500 K) is regarded as natural daylight and colour colours beyond these values are regarded as coolish bluish white light [Wikipedia, November 2022].

### High Thermal and Chemical Stabilities

Beside the physical properties of these materials (CRI, CCT and luminescence efficiencies) mentioned above, white light emitting or persistent luminescent phosphors, need to be chemically and thermally stable. This means that they need to withstand temperature chemical changes. Phosphor materials having such characteristics are phosphates, silicates, and aluminates.

### Appropriate Choice of Host Compounds and Dopants

Host materials by their very nature do not produce light emissions, hence the need to choose a dopant or co-dopant that could act as luminescent centers in the host material. Shrivastava et al. [4] has identified several host materials that could be doped with Dy^3+^ ions for white colour emissions. Silicate and aluminate host compounds appear to take the lead in this regard because much of their properties relate to prolonged emissions long after the removal of the UV excitation source. Other researchers have focused on phosphates as host materials for the embedding of the Dy^3+^ ion dopant to produce enhanced white colour luminescence. Besides white colour emissions produced from Dy^3+^ doped host materials, other properties such as persistent luminescence has also been observed when Dy^3+^ ion is co-doped with other rare earth ions. Aluminates has gained much popularity as a host material because of its high chemical stability, high quantum yield, as well as a wide band gap of around 6 eV [6]. Silicates, likewise, have similar properties; they have a high chemical stability, a high quantum yield and have a wide band gap and is the most widely used host material for persistence luminescence [6]. On the other hand, phosphates have a relatively large band gap, possess different crystallographic structures, and display excellent luminescence properties but have lower persistent luminescence property compared to silicates and aluminates. These host materials show enhanced long afterglow effects when codoped with Eu^2+^ ions. Besides using Dy^3+^ as a dopant, others have considered Eu^3+^, Sm^3+^, Ce^3+^ as compatible dopant pairs in the above-mentioned host materials. Single-phased host materials are important because they are cost effective in their fabrication as compared to using 2 or 3 phosphor materials to produce the same effect.

### Nature of Y/B Intensity Ratios in Producing Electric Dipole (ED) or Magnetic Dipole (MD) Transitions

It is noted that some Dy^3+^ doped phosphor materials experience either electric dipole or magnetic dipole transitions. When yellow colour emissions (ED) are higher than the blue colour emissions (MD), then the intensity Y/B ratio is greater than 1, but if the blue colour emission is higher than the yellow emissions, then the peak intensity Y/B emission ratio is less than 1.

### Emission Lifetimes: Fluorescence or Phosphorescence Emissions

Fluorescence electronic transitions happens almost instantaneously (t < 10^− 8^s) without changes in its spin state, but phosphorescence emissions are much slower (t > 10^− 8^s) and is accompanied by changes in its electronic spin states [7]. Extensive research is being done to find Dy^3+^ doped or co-doped phosphor materials that exhibits persistent luminescence long after the excitation source is removed. In this regard silicates and aluminates are promising candidates for such endeavors.

### Influence of Concentration on the Y/B Ratio in Dy^3+^ Doped Materials

If Dy^3+^ ion has the same valency as the cation (e.g. Y^3+^), then the intensity ratio Y/B will hardly change with increases in concentration. On the other hand, if Dy^3+^ ion has a different valency than the cation (e.g. Y^2+^) then the Y/B intensity ratio will change with increases in concentration [5] but not so with temperature changes.

### Mechanism of White Light Colour Emission from Dy^3+^ Doped Host Materials

The energy level diagram below is taken from the paper of Shamal et al. [8], shows the energy transfer mechanism of a typical Dy^3+^ doped host material. This will typically happen when Dy^3+^ is doped with aluminates, phosphate, or silicate host materials, since the ion will be positioned within the band gap of these materials. It should be noted that either the blue light or yellow light emission could be more prominent dependent on whether Dy^3+^ ions are positions within the environment with inversion or without inversion symmetry, thus affecting the nature of the white light produced.

**Fig. 1:**
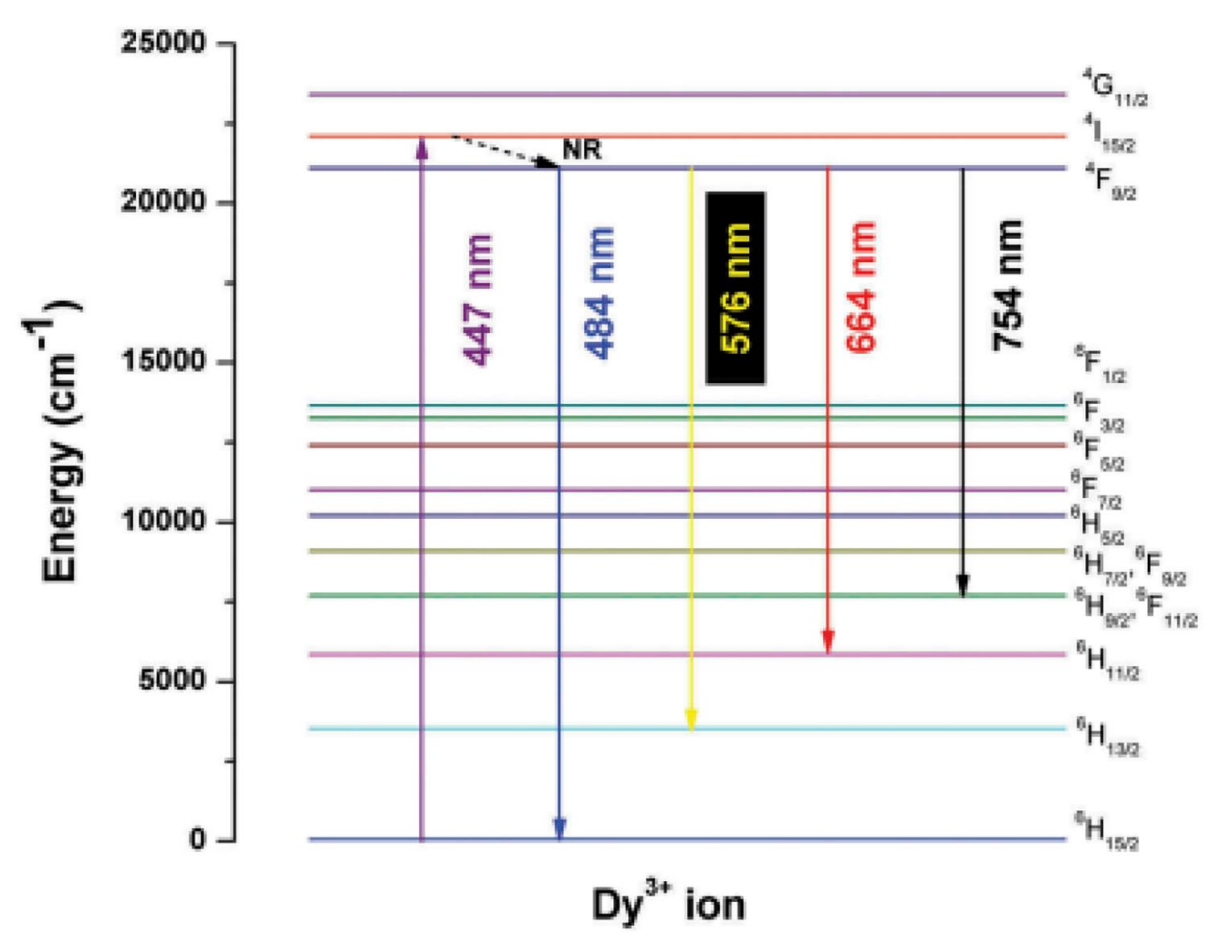
Initially, Dy^3+^ ion is excited using an excitation wavelength of 447 nm. The excited electron moves from the ground state (^6^H_15/2_) of Dy^3+^ ion to its upper excited state (^4^I_15/2_). It then relaxes non-radiatively to the metastable ^4^F_9/2_ state, populating it and thereafter makes various downward transitions, resulting in different colour emissions. The emission at wavelength 484 nm corresponds to blue light (^4^F_9/2_ → ^6^H_15/2_), a yellow colour emission occurs at 576 nm (^4^F_9/2_ → ^6^H_15/2_), a red colour emission occurs at 664 nm (^4^F_9/2_ → ^6^H_11/2_) and finally a brown colour emission occurs at 754 nm (^4^F_9/2_ → ^6^H_9/2_, ^6^F_11/2_). Appropriate ratios of Yellow to Blue will result in white colour light. When Dy^3+^ is codoped with other rare earth ions, the mechanism of such is discussed at a later stage in this paper

## White light Emissions from Singly Doped Dy^3+^ Ions Corresponding to Magnetic or Electric Dipole Transitions from Phosphate Type Materials

Several research papers [9–15] have been published with Dy^3+^ as a dopant ion, corresponding to magnetic dipole transitions, as shown in Table 1(a). For all these papers the common synthesis method is the solid-state reaction method. The excitation wavelength for all these phosphor materials falls in the UV range from 348 to 387 nm. The PL peak intensities, corresponding to blue emissions is situated in the narrow wavelength range of 480 to 487 nm, while that for yellow PL emissions were found in the 571 to 577 nm wavelength range. As far as the Y/B intensity ratio is concerned, they are all less than 1, implying that the blue light emissions dominate over the yellow colour emissions. This further implies that Dy^3+^ ions are located at high symmetry sites with inversion center in the environment surrounding the phosphate host material [12]. The CCT values calculated for these Dy^3+^ doped phosphate materials fall in the cool bluish white colour range, with temperature values in the range from 5962 to 12,606 K. Phosphates of this nature are mainly adaptable for outdoor settings. In all cases, the Y/B values appear to change with concentrations, implying that Dy^3+^ ions are displaced by ions of different valences (Sr^2+^, Ca^2+^, Na^+^), leading to the creation of defects in the crystal lattice.

**Table 1(a):** Magnetic dipole transitions from singly doped Dy^3+^ phosphate materials

No	Sample	Method of synthesis	UV excitation wavelength (nm)	PL emission wavelength (nm)		CIE coordinates	CCT (K)	Ref
				Blue	Yellow			
1	Sr_3_Y(PO_4_)_3_	Solid state	350	484	576	(0.310, 0.332)	6632	[9]
2	NaCaPO_4_	Solid state	376	482	575	(0.320, 0.370)	5962	[10]
3	CaZr_4_(PO_4_)_6_	Solid state	350	487	577	(0.279, 0.306)	9280	[11]
4	NaSrPO_4_	Solid state	348	481	573	(0.298, 0.342)	7186	[12]
5	Sr_3_Gd(PO_4_)_3_	Solid state	387	483	575	(0.252, 0.294)	12,606	[13]
6	Ca_9_Bi(PO_4_)_7_	Solid state	350	487	577	(0.300, 0.358)	6919	[14]
7	NaLa(PO_3_)_4_	Solid state	350	485	571	(0.292, 0.335)	7592	[15]

Besides the magnetic dipole transitions observed from singly Dy^3+^ doped phosphate materials, Table 1(b) also displays electric dipole transitions associated with these materials [16–20]. Likewise, the solid- state reaction method was the common method of synthesis of these materials. The excitation wavelength for these materials is a wide range from 325 to 410 nm. The PL emission peak for the less dominant blue colour ranged from 480 to 496 nm, while the dominant yellow colour peak ranged from 573 to 576 nm. The dominant hypersensitive yellow colour emissions is due to Dy^3+^ ions being located at low symmetry sites without inversion. The CIE coordinates, which was determined from the PL intensity peaks, was found to produce CCT values in the range from 3707 to 6799 K, which is typically in the warm to cool blue to white colour regions. This range of temperatures is ideal for energetic environmental settings, promoting alertness and sharpness in various indoor or outdoor activities. The intensity Y/B ratios in all cases is greater than 1 for electric dipole transitions and whose values changes with concentrations, implying that Dy^3+^ ions are displaced by ions of different valences (Ba^2+^, K^+^, Na^+^, Ca^2+^), thereby creating defects in the host matrix. The warm white light produced in most of this phosphate doped materials could be considered for commercialization when excited by UV light.

**Table 1(b):** Electric dipole transitions from singly doped Dy^3+^ doped phosphate materials

No	Sample	Method of synthesis	UV excitation wavelength (nm)	PL emission wavelength (nm)		CIE coordinates	CCT (K)	Ref
				Blue	Yellow			
1	Ba_3_Y(PO_4_)_3_	Solid state	348	486	575	(0.347, 0.381)	4996	[16]
2	KLa(PO_3_)_4_	Solid state	325	480	573	(0.308, 0.326)	6799	[17]
3	Na_3_MgZr(PO_4_)_3_	Solid state	365	487	576	(0.403, 0. 416)	3707	[18]
4	Ca_19_Mg_2_(PO_4_)_4_	Solid state	365	483	572	(0.400, 0.452)	3994	[19]
5	Na_3_Al_2_P_3_O_12_	Solid state	410	486	575	(0.442, 0. 454)	3279	[20]

## White Light Emissions from Co-Doped Dy^3+^ Ions Corresponding to Magnetic or Electric Dipole Transitions from Phosphate Type Materials

In the case of co-doped Dy^3+^ phosphate host materials, the mechanism of white light emission is different from that of singly doped phosphor materials, as seen in Table 1(c) [21, 22]. The difference emanates from type of co-dopant used and the role it plays, either as an activator or a sensitizer in the host compound. If Ce^3+^ ion is used as a dopant, it can effectively enhance the luminescence characteristics of the phosphate material, because of it has a broad and intense emission band due to its parity and spin allowed 4f -5d transition and which is electric dipole in nature [23]. The effect of this is to boost the parity forbidden nature of the Dy^3+^ transitions [24]. On the other hand, the addition of Sm^3+^ as a co-dopant will play a crucial role in addressing the shortage of the red ingredient of white light [21]. One can say that the white light emission from singly doped Dy^3+^ ions have drawbacks in producing light emissions with a low CRI and a high CCT value [21]. Thus, Sm^3+^ as a co-dopant can ameliorate this situation by producing high quality white light of a high CRI and a low CCT value. Further, the addition of Eu^3+^ as a co-dopant, exhibits strong red colour emissions and is a useful ingredient for high quality white light production. In this regard it can be said that the addition of Eu^3+^ as a co-dopant is effective in producing better warm colour white light [22]. In the case of co-doped xDy^3+^ /yEu^3+^ (where x = 3 mol % and y = 2, 4, 6, 8 and 10 mol**%**) NaSrPO_4_ phosphor material, the white light produced will vary for each pair of CIE coordinates, as shown in Table 1(c) [22]. For this example, the intensity of the Dy^3+^ ion is kept constant at 3 mol% while the concentrations of Eu^3+^ is varied from 2 mol% to 10 mol%. From the PL emission spectra, it is observed that the blue and yellow emissions of Dy^3+^ ions decrease for increases in the concentration of Eu^3+^ ions. This implies that energy is being effectively transferred from Dy^3+^ ions to Eu^3+^ ions. The CIE as well as the CCT values are shown in Table 1(c) for such variations.

**Table 1(c):** Magnetic or electric dipole transitions from Dy^3+^ co-doped phosphate materials

No	Sample	Method of synthesis	UV excitation wavelength (nm)	PL emission wavelength (nm)			CIE coordinates	CCT (K)		Colour	Ref
				Blue	Yellow						
1	Ca_3_(PO_4_)_2_: xDy^3+^/yEu^3+^	Hydrothermal	362	481	573		x = 3 & y = 2 (0.295, 0.330)	7522		Bluish white	[21]
							x = 3 & y = 4 (0.320, 0.334)	6096		Bright white	
							x = 3 & y = 6 (0.329, 0.326)	5665		Bright white	
							x = 3 & y = 8 (0.346, 0.324)	4858		Bluish white	
							x = 3 & y = 10 (0.367, 0.326)	4022		Warm white	
				Blue		Yellow					
2	NaSrPO_4_: xDy^3+^/ySm^3+^	Sol-gel	348	484		577	x = 2 & y = 0 (0.3408, 0.3722)		5129	Cool white	[22]
							x = 2 & y = 1 (0.3592, 0.3697)		4506	Neutral white	
							x = 2 & y = 2 (0.3821, 0.4084)		4100	Neutral white	
							x = 2 & y = 3 (0.3893,0.4197)		3989	Warm white	

From the above table, the maximum PL emission occurs for the phosphor material having CIE coordinates (0.295, 0.330) yet its CCT value is high and is not ideal for commercialization, but with concentration tuning its CIE and CCT values was adjusted to a warm white light, as can be seen in Table 1 (c). Thus, co-doping phosphate host materials can be an effective way of tuning light emission to the desired white colour light as compared to singly doped Dy^3+^ ion. As with singly doped Dy^3+^ ions, the excitation and emission wavelengths are in a similar range. For these samples, blue light emission was more dominant than the yellow colour emission and its CCT values were tuned with concentration variations. The preparation methods for these samples varied from hydrothermal to sol-gel methods of synthesis. Whilst these two samples provide compelling reasons for co-doping and tuning with concentration, other researchers have focused on optimizing the x and y values when obtaining their CIE and CCT values [23, 24], as seen in Table 1(d). When Ca_3_Mg_3_(PO_4_)_4_ is doped with Dy^3+^ ions (Li^+^ is a compensator ion) only, the CIE value at optimum concentration is (0.265, 0.339), which corresponds to a CCT value of 9536 K. Further, in co-doping Ca_3_Mg_3_(PO_4_)_4_ with Ce^3+^ ions, with Ce^3+^ ion concentration being fixed while the concentration of Dy^3+^ was varied, then at optimal values of these concentrations, their CIE value changed to (0.317, 0. 393), corresponding to a CCT value of 4739 K. In co-doping, we observe 2 fundamental changes; in the first case, we observe magnetic dipole transition, and secondly when co-doped with Ce^3+^ ions, the transition changed from magnetic dipole to electric dipole transition. In this scenario the Y/B value changed from 0.9 to 1.265 brought about by co-doping the material with Ce^3+^ ions. Further the cool white colour produced results from the energy transfer from Ce^3+^ ion to Dy^3+^ ions. Combustion synthesis method was used here and besides the two typical emission wavelengths for blue and yellow lights, the excitation wavelength for this sample was at 316 nm.

**Table 1(d):** Magnetic or electric dipole transitions from Dy^3+^ coped phosphate materials

No	Sample	Method of synthesis	UV excitation wavelength (nm)	PL emission wavelength (nm)		CIE coordinates	CCT (K)	Colour	Ref
				Blue	Yellow				
1	Ca_2_Mg_3_(PO_4_)_4_: Dy^3+^/Li^+^	Combustion	316	482	572	(0.265, 0.339) [MD]	9536	Bluish white	[23]
	Ca_3_Mg_3_(PO_4_)_4_: xCe^3+^/yDy^3+^/Li^+^	Combustion	316	482	572	(0.317, 0.393) [ED]	4739	Cool white	[23]
2	Zn_2_P_2_O_7_:xCe^3+^/yDy^3+^	Solid state	290	479	572	(0.276, 0.287) [ED]	10,490	Cool bluish white	[24]

Similarly, in co-doping Zn_2_P_2_O_7_ with Ce^3+^ ions have produced enhanced white emissions in the bluish region due to the energy transfer from Ce^3+^ ions to Dy^3+^ ions. This transition is due to the magnetic dipole interactions between the Dy^3+^ ions. At optimal concentration of Ce^3+^ (x fixed) and Dy^3+^ (y varied) ions, the CIE value obtained was (0.2764, 0.2876), which corresponds to a CCT value of 10,490 K, which is a coolish blue, white light. The excitation wavelength applied is a deep UV-B 290 nm wavelength, where the solid- state reaction method was used for preparing these samples.

## White Light Emissions from Singly Doped Dy^3+^ Ions with Silicates as Host Materials

The next category of host materials for consideration in white light emission is through doping Dy^3+^ ions with silicates. Silicates are easy to prepare and have excellent thermal and chemical stability [25]. The role of silicates in this scenario is for the generation of phosphorescence white light (t > 10^− 8^s) instead of fluorescence white light (t < 10^− 8^s) [4]. When Dy^3+^ ions are embedded in silicate host materials, it creates traps, which prolongs the emission of white light due to the continuous energy transfer from these traps to luminescent centers [4]. Several research papers [26–30] have been considered in respect of singly doped Dy^3+^ silicate materials, as seen in Table 2(a). The most popular synthesis route is the solid-state reaction method, followed by the combustion synthesis route of preparation. The excitation wavelengths have a wide range from 314 to 352 nm.

**Table 2(a):** Emissions from singly doped Dy^3+^ silicate phosphor materials

No	Sample	Method of synthesis	UV excitation wavelength (nm)	PL emission wavelength (nm)		CIE coordinates	CCT (K)	Colour	Ref
				Blue	Yellow				
1	CaMgSi_2_O_6_	Solid state	352	481	570	(0.320, 0.358) [ED] > 1	6013	Cool white	[26]
2	Ba_2_Al_2_SiO_7_	Solid state	350	481	575	PL: (0.319, 0.365) TL: (0.319, 0.365) [MD] < 1	6034 6034	Cool white	[27]
3	Sr_2_MgSi_2_O_7_	Solid state	351	480	575	PL: (0.33, 0.35) TL: (0.33, 0.37) [MD] < 1	5601 5592	Cool white	[28]
4	Sr_2_SiO_4_	Combustion	314	478	574	PL: (0.214, 0.264) TL: (0.282, 0. 279) [MD] < 1	25,571 10,202	Bluish white	[29]
5	L_i2_SrSiO_4_	Solid state	250	478	572	(0.263, 0.292) [MD] < 1	11,615	Bluish white	[30]

Typical emissions bands in the blue (470–480) nm and yellow (570–575) bands were observed for these persistent or prolonged white light emissions. The optimal intensity Y/B ratios are likewise dependent on the crystal field in which the Dy^3+^ ions are located. For these samples we notice that the Y/B values varies with concentrations due to Dy^3+^ having different valences compared to the cations (Sr^2+^, Ba^2+^, Li^+^). The calculated CCT values were found to range from 5592 to 25,571 K, which makes these silicate materials ideal for outdoor activities, especially when we see cool bluish white colour emissions. Whilst the role of Dy^3+^ ions is for creating traps in host materials for prolonged afterglow effects, no material has ever been commercialized because of its lack of consistency in its afterglow effects and due to the degradation of the light with time [28]. These samples comprise of a mixture of electric dipole and magnetic dipole transitions as can be seen in the table. CIE was determined from both PL and TL measurements in some cases. The CIE differences between these two measurements are relatively small. The CCT values for these silicate materials fall in the cool bluish white color region. Most of these materials produce prolonged white colour emissions, with the highest being more than 1 h for the Dy^3+^ doped SrSiO_3_ silicate phosphor material, not displayed in the table.

For the SrSiO_3_ phosphor material, its long white light phosphorescence is due to the low trap depth that was created at 0.59 eV in the band gap of the host [4, 31]. Another silicate material that also showed a long phosphorescence white luminescence was the Sr_2_MgSi_2_O_7_ phosphor material with a decay time of 40 min after the UV excitation source was removed [4, 28]. Another silicate, also not mentioned in the table but in the review of Shrivastava et al. [4, 32], shows that another silicate Ca_2_MgSi_2_O_7_ doped with Dy^3+^ and with similar emission characteristics as those in Table 1 (d), but with an excitation wavelength of 254 nm, revealed a huge afterglow of 3 h and is the most preferred single doped silicate material. The decay time for all these single Dy^3+^ doped silicate materials show afterglow times ranging from milliseconds to a huge 3 h.

## Phosphorescence White Light Emissions from Singly Doped Dy^3+^ Ions in Aluminate Host Materials

Dy^3+^ doped aluminate phosphor materials are shown in Table 2 (b). Several research papers [33–37] have been studied in respect of Dy^3+^ doped aluminate host materials, which were mostly prepared by the solid- state reaction method. The excitation wavelength has a narrow UV range from 348 to 351 nm. Typical peak emissions of blue colour were found around the 481 to 492 nm wavelength band, as well as yellow colour emissions around the 574 to 581 nm wavelength band. Magnetic dipole transitions were observed for aluminates referenced as [33], [34] and [37], while electric dipole transitions are referenced as [35] and [36], respectively. The intensity Y/B peak ratio of these samples were also found to vary with Dy^3+^ concentrations (Sr^2+^, Ca^2+^, Ba^2+^) due to the creation of defects. The Y/B value for sample referenced as [36], with similar valency as Dy^3+^, showed no variation with concentration but variation was observed with temperature changes. In the case of sample referenced [37], we see a change in the magnetic dipole to electric dipole transitions for variations in Dy^3+^ concentrations. CIE values were not calculated for all aluminate samples, but for those that were calculated, revealed phosphorescence white colour emissions with CCT values ranging from 3784 to 5951 K. These temperatures reflect warm to cool bluish white phosphorescence light emissions for selective samples. It is of interest to note that the CaAl_2_O_4_ [34] sample revealed prolonged phosphorescence white light emissions of up to 115 min.

**Table 2(b):** Emissions from singly Dy^3+^ doped aluminate materials

No	Sample	Method of synthesis	UV excitation wavelength (nm)	PL emission wavelength (nm)		CIE coordinates	CCT (K)	Y/B	Ref
				Blue	Yellow				
1	Sr_2_MgAl_2_O_6_	Solid state	348	496	581	None	None	[MD] < 1	[33]
2	CaAl_2_O_4_	Sol-gel	350	482	574	None	None	[MD] < 1	[34]
3	Ba_2_YAlO_5_	Solid state	357	492	580	(0.396, 0. 401)	3784	[ED] > 1	[35]
4	YAlO_3_	Sol-gel combustion	351	487	578	(0.327, 0. 314)	5787	[ED] > 1	[36]
5	Ba_5_CaAl_4_O_12_	Solid state	348	481	574	(0.322, 0.349)	5951	[ED] and [MD]	[37]

## Co-Doped Dy^3+^ Silicate and Aluminate Phosphor Materials for Phosphorescence White Light Emissions

While singly doped Dy^3+^ ions with silicate and aluminate phosphor materials are reasonably good for phosphorescence of white light emissions, the role of co-dopants ions could play a slightly different role in the choice of the dopant ions, which may or may not produce white light. Thus, for persistent white light (or other colours), it will depend on the role of Dy^3+^ ions in the silicate and aluminate host materials. Finding appropriate host materials for persistent luminescence is a narrow field, thus posing challenges for modern day researchers. For a long time, the ZnS: Cu phosphor material has dominated the market in terms of its persistent luminescence emissions until the emergence of more rigorous and resilient phosphor materials such as silicates and aluminates. In 1966, bright green luminescence emissions were observed in the SrAl_2_O_4_:Eu^2+^ [38] phosphor material and since then research has evolved in many ways yet with no commercialization to date. Later in 1996, Matsuzawa [39] co-doped this material Dy^3+^ and found long lasting afterglow by UV excitation and ever since more than 100 papers were published. Then came Lin et al. [32] in 2001, who fabricated Sr_2_MgSi_2_O_7_:Eu^3+^/Dy^3+^ phosphor material and found yet again prolonged afterglow for a silicate material. Now 25 years later, after the discovery of SrAl_2_O_4_:Eu^2+^/Dy^3+^ the search for persistent luminescent materials is still ongoing but at a snail’s pace. These two materials, SrAl_2_O_4_(aluminate)and Sr_2_MgSi_2_O_7_ (silicate) have dominated the research arena for decades until a novel long lasting and persistent (consistently) luminescence material is proposed for phosphorescence emissions. Another, aluminate material in the family of alkaline earth metals, MAl_2_O_4_ (M = Ca, Sr, Ba) has received considerable attention due to its persistent phosphorescence characteristics [7]. The aluminate Sr_4_Al_14_O_25_:Eu^2+^/Dy^3+^ [40] phosphor material, has produced prolonged phosphorescence luminescent emissions of up to 20 h. There is no consensus from all the research thus far, whether co-doping has impacted on the afterglow effects. Further, synthesis methods have proven to be crucial in afterglow effects, with the solid-state reaction method leading the race, followed by the combustion method. The role of Dy^3+^ ions is merely to create traps, particularly low traps of about 0.65 eV in the host materials, that would be ideal for phosphorescence luminescence [38].

Many research papers of co-doped Eu^2+^/Dy^3+^ aluminates have been analyzed in the review paper of Eeckhout et al. in 2010 [38] for materials that show persistent luminescence long after the UV excitation source is removed. The following aluminates, with afterglow times given in brackets, shows the prominent role played by aluminates, and they are: SrAl_2_O_4_:Eu^2+^/Dy^3+^ [38, 41] (more than 30 h), BaAl_2_O_4_:Eu^2+^/Dy^3+^ [38, 42] (more than 2 h), Sr_4_Al_14_O_25_:Eu^2+^/Dy^3+^ [38, 43] (more than 20 h), SrAl_4_O_7_:Eu^2+^/Dy^3+^ [38, 44] (more than 3 h), SrAl_12_O_19_:Eu^2+^/Dy^3+^ [38, 45] (more than 3 h) and SrMgAl_10_O_17_ [38, 46] (more than 3 min).

What was interesting from this review paper was that when BO_4_ was incorporated [47] into SrAL_2_O_4_:Eu^2+^/Dy^3+^, the trap depth appears to have decreased from 0.79 eV to 0.65 eV, allowing for prolonged phosphorescence luminescence.

Further in the review paper of [38], prolonged phosphorescence properties of silicate co-doped materials are discussed. The after-glow times of these silicate materials are given in brackets: SrMgSi_2_O_7_:Eu^2+^/Dy^3+^ [38, 48] (more than 10 h), SrMgSi_2_O_8_:Eu^2+^/Dy^3+^ [38, 49] (more than 10 h), Ca_3_MgSi_2_O_8_:Eu^2+^/Dy^3+^ [38, 50] (more than 5 h), Ba_3_MgSi_2_O_8_:Eu^2+^/Dy^3+^ [38, 50] (more than 1 h), CaMgSi_2_O_6_:Eu^2+^/Dy^3+^ [38, 51] (more than 4 h) and Sr_2_Al_2_SiO_7_:Eu^2+^/Dy^3+^ [38, 52] (more than 2 h).

## Mechanism of Energy Transfer of Dy^3+^ Codoped Phosphor Host Materials

Many models have been postulated since 1996 to explain the mechanism of persistent luminescence in many of the above host materials but none have a uniform strategy on the precise mechanism of energy transfer. Besides the accepted theoretical model of charges being excited and getting caught in “trap states” within the band gap of host materials, and after thermal stimulation, they get “detrapped” leading to the afterglow effects observed for this selective class of materials. About two decades of research has been done on the first SrAl_2_O_4_:Eu^2+^, Dy^3+^ phosphor material for prolonged afterglow effects (more than 30 h). The first ever model on energy transfer was proposed by **Matsuzawa et al.** [39] in 1996. They proposed that holes be the prominent charge carrier. For the selective SrAl_2_O_4_:Eu^2+^, Dy^3+^ phosphor material, they indicated that when Eu^2+^ is excited, holes escape to the valence band, leaving behind a monovalent Eu^+^ charge, which is captured by the Dy^3+^ ion within the band gap of the host, creating a tetravalent Dy^4+^ ion charge. After thermal stimulation (kT), the trapped hole then migrates back to the valence band and back to the monovalent Eu^+^ ion, this process can be regarded as return to the ground state of the divalent Eu^2+^ ion, through emission of a photon that accounts for the afterglow effects. This process could similarly be explained with direct transition within the band gap through recombination of electron-hole effects [34]. Later in 2003, **Aitasalo et al.**. [53] used a non Dy^3+^ doped CaAl_2_O_4_:Eu^2+^, Nd^3+^ sample to explain persistent luminescence, they proposed that electrons escape directly from the valence band of the host to various unknown traps within the band gap region, a hole is created from this process in the valence band. This hole then moves within the valence band where it is captured by a calcium vacancy in the band gap region. By thermal stimulation (kT), the electrons from various trap levels then migrate to an oxygen vacancy, then by recombination with the hole calcium site, energy is released and whose energy is then used to excite Eu^2+^ from its ground state to the 5d^1^ state of Eu^2+^ ion, which is then de-excited to its ground with an emission of a photon [34, 53]. Later in 2005, **Doren et al.** [54] proposed their method for persistent luminescence, using the SrAl_2_O_4_:Eu^2+^, Dy^3+^ phosphor material but refuted claims made by Aitasalo et al. They stated that electrons are excited from the ground state of Eu^2+^ ion to its upper excited 5d^1^ state, and because they are so close to the conduction band, which then allows for easy migration into the conduction band, after which they are trapped by a trivalent ion such as Dy^3+^ ion, creating a divalent ion. Thermal excitation releases this trapped electron which then moves to the conduction band and then recombines with the luminescent 5d^1^ state and after de-excitation moves to the ground state of Eu^2+^ with an emission of a photon [34]. Later, at the same time, another model was proposed by **Clabau et al.** [55] on the same material as above, appears to be very simplistic in nature. Firstly, an electron is excited from the ground state of Eu^2+^ ion to its upper excited state 5d^1^. This electron then migrates to an oxygen vacancy (defect site) and after thermal stimulation, the electron then moves back to its luminescent center at 5d^1^ and after de-excitation moves back to its ground state with an emission of a photon (without moving along the conduction band, as in the case of Doren et al’s model).

Some models proposed currently are from two research groups, one by **Castaing et al.** [56] and another by **Tanaka et al.** [57], both published in 2021, shows departures from the earlier models proposed a decade ago on the same SrAl_2_O_4_:Eu^2+^, Dy^3+^ phosphor material. In the case of the Tanaka et al. model, electrons are first excited from the ground state of the Eu^2+^ ion to excited energy levels just below the conduction band, and through migration into the conduction band, they are then captured by “trap” levels below the conduction band. By thermal stimulation, they then make another migration (detrapping) through the conduction band back to the luminescent center and then are de-excited to the ground state of Eu^2+^ ion (recombination of electron-hole) though the release of a photon of energy. This model is very simplistic in nature as well. The work of Castaing et al., is a simple process whereby charges are excited to the 5d^1^ energy level of Eu^2+^ ion from its ground state, which lies just below the conduction band of the host material. This electron is then trapped at the storage site (charge trapping) and by thermal excitation, it is released back to the luminescent center (5d^1^) and is then de-excited to its ground state through the release of a photon of energy.

## Conclusion

For Dy^3+^ ions, two separate transitions; namely, a blue ^4^F_9/2_ →^6^H_15/2_ and a yellow ^4^F_9/2_→ ^6^H_13/2_ are responsible for white colour emissions, if the Y/B intensity ratios are carefully optimized. However, the role of Dy^3+^ ions is not only for white light emissions for LED applications, but it can also play a fundamental role in persistent luminescence when co-doped with dopants pairs such as Eu^2+^, Eu^3+^, Ce^3+^ ions. Due to the lack of the red colour ingredient of white light, red emitting dopants such as Sm^3+^ or Eu^3+^ have played a huge role in ameliorating the situation. Many factors must be taken into consideration in producing enhanced white luminescence for prolonged colour emissions. Factors such as cost effectiveness, chemical and thermal stability, appropriate choice of host compounds and choice of dopants should be on the top of the list in this regard. In respect of these factors, the position of the Dy^3+^ ions in the host material, either at symmetric or asymmetric locations in the host compound, will determine the nature of the transitions as well as its CIE and CCT values. Several host materials have been considered in this respect and they range from phosphates to silicates and aluminates mainly because of their excellent chemical and stability characteristics. In respect of singly doped Dy^3+^ ions in phosphate host materials, the dominant blue magnetic dipole transitions in different host materials produces white light whose CCT values are generally more than 6000 K, making it ideal for energetic outdoor settings. On the other hand, white colour emissions due to electric dipole transitions (yellow colour dominant), their CCT values are in the range from 3000 to 6800 K, making it ideal for both indoor (warm) and outdoor (cool bluish white) settings. The situation is quite different when Dy^3+^ ions are co-doped with different phosphate materials. When co-doped with Eu^3+^ ions in the phosphate host, it appears that their CIE and CCT values are tuned with concentration changes, which mainly results from the energy transfer from Dy^3+^ ions to Eu^3+^ ions. An analogous situation was observed when Dy^3+^ ions was co-doped with Sm^3+^ ions. However, something strange happens when Dy^3+^ ions is co-doped with Ce^3+^ ions. In this scenario, we observe a change in the dipole transition from magnetic to electric dipole transition when comparisons are made with singly doped Ca_3_Mg(PO_4_)_4_:Dy^3+^ to co-doped Ca_3_Mg(PO_4_)_4_:Ce^3+^/Dy^3+^ ions, resulting in the energy transfer from Ce^3+^ ions to Dy^3+^ ions. The CCT values for white colour emission drops when the magnetic dipole transition moves over to the electric dipole transition from this co-doping effect. The search for persistent luminescent materials has led us to exclusively to study two types of host materials; namely, silicates and aluminates, which has been extensively studied in literature. The role of the Dy^3+^ ions in this scenario is in the creation of energy traps within the band gap of the host material. Since the discovery of SrAl_2_O_4_:Eu^2+^/Dy^3+^ and Sr_2_MgSi_2_O_7_:Eu^2+^/Dy^3+^ phosphor materials for prolonged luminescence, the search for better materials is just around the corner. Singly doped Dy^3+^ silicate materials appear to emit white light ranging from milliseconds to more than an hour for SrSiO_3_ and to a high 3 h for the Ca_2_MgSi_2_O_12_ phosphor material. Whilst these transitions are comprised of both magnetic and electric dipole transitions, their CCT values are above 5500 K and indicative of prolonged luminescence for outdoor settings. Whilst not much pertinent information is given about single doped aluminates, they appear to have their CCT values to be less than 6000 K and more than adequate for cool to warm white lighting for indoor settings. As far as phosphorescence white light emissions in aluminates are concerned, only CaAl_2_O_4_ has revealed prolonged luminescence of more than 115 min. On the other hand, aluminates and silicates are co-doped with other rare –earth ions (such as Eu^2+^), we notice enhanced phosphorescence of up to 30 h for the SrAl_2_O_4_:Eu^2+^/Dy^3+^ phosphor material and 20 h for another aluminate Sr_4_Al_14_O_25_:Eu^2+^/Dy^3+^ phosphor material, with others having a decay time of at least an hour. This points to the fact the intricate role of Eu^2+^ when doped with Dy^3+^ ions in producing persistent phosphorescence.

## Data Availability

N/A.
